# Multiparameter body composition analysis on chest CT predicts clinical outcomes in resectable non-small cell lung cancer

**DOI:** 10.1186/s13244-025-01910-0

**Published:** 2025-02-06

**Authors:** Yilong Huang, Hanxue Cun, Zhanglin Mou, Zhonghang Yu, Chunmei Du, Lan Luo, Yuanming Jiang, Yancui Zhu, Zhenguang Zhang, Xin Chen, Bo He, Zaiyi Liu

**Affiliations:** 1 Guangdong Cardiovascular Institute, Guangdong Provincial People’s Hospital, Guangdong Academy of Sciences, Guangzhou, Guangdong China; 2 Department of Radiology, Guangdong Provincial People’s Hospital (Guangdong Academy of Medical Sciences), Southern Medical University, Guangzhou, Guangdong China; 3 Guangdong Provincial Key Laboratory of Artificial Intelligence in Medical Image Analysis and Application, Guangzhou, Guangdong China; 4 Department of Medical Imaging, The First Affiliated Hospital of Kunming Medical University, Kunming, Yunnan China; 5 Department of Intensive Care Unit, The First Affiliated Hospital of Kunming Medical University, Kunming, Yunnan China; 6 Department of Radiology, Guangzhou First People’s Hospital, School of Medicine, South China University of Technology, Guangzhou, Guangdong China

**Keywords:** Lung cancer, Body composition, Disease-free survival, Overall survival, Computed tomography

## Abstract

**Objectives:**

This study investigates the association between baseline CT body composition parameters and clinical outcomes in patients with resectable non-small cell lung cancer (NSCLC).

**Methods:**

Patients who underwent surgical resection for NSCLC between January 2006 and December 2017 were retrospectively enrolled in this multicenter study. Body composition metrics, including the area of skeletal muscle, intermuscular adipose tissue, subcutaneous adipose tissue, visceral adipose tissue, muscle radiodensity, and derivative parameters from five basic metrics mentioned before, were calculated based on preoperative non-contrast-enhanced chest CT images at L1 level. The Cox proportional hazards regression analysis was used to evaluate the association between body composition metrics and survival outcomes including overall survival (OS) and disease-free survival (DFS).

**Results:**

A total of 2712 patients (mean age, 61.53 years; 1146 females) were evaluated. A total of 635 patients (23.41%) died. 465 patients (19.51%) experienced recurrence and/or distant metastasis. After multivariable adjustment, skeletal muscle index (SMI, HR = 0.86), intermuscular adipose index (IMAI, HR = 1.49), and subcutaneous adipose index (SAI, HR = 0.96) were associated with OS. Similar results were found after stratification by gender, TNM stage, and center. There was no significant association between all body composition metrics and DFS (all *p* > 0.05). The body composition metrics significantly enhance the model including clinicopathological factors, resulting in an improved AUC for predicting 1-year and 3-year OS, with AUC values of 0.707 and 0.733, respectively.

**Conclusions:**

SMI, IMAI, and SAI body composition metrics have been identified as independent prognostic factors and may indicate mortality risk for resectable NSCLC patients.

**Critical relevance statement:**

Our findings emphasize the significance of muscle mass, quality, and fat energy storage in clinical decision-making for patients with non-small cell lung cancer (NSCLC). Nutritional and exercise interventions targeting muscle quality and energy storage could be considered for patients with NSCLC.

**Key Points:**

- Multiparameter body composition analysis is associated with the clinical outcome in NSCLC patients.
- Assessing muscle mass, quality, and adipose tissue helps predict overall survival in NSCLC.
- The quantity and distribution of body composition can contribute to unraveling the adiposity paradox.

**Graphical Abstract:**

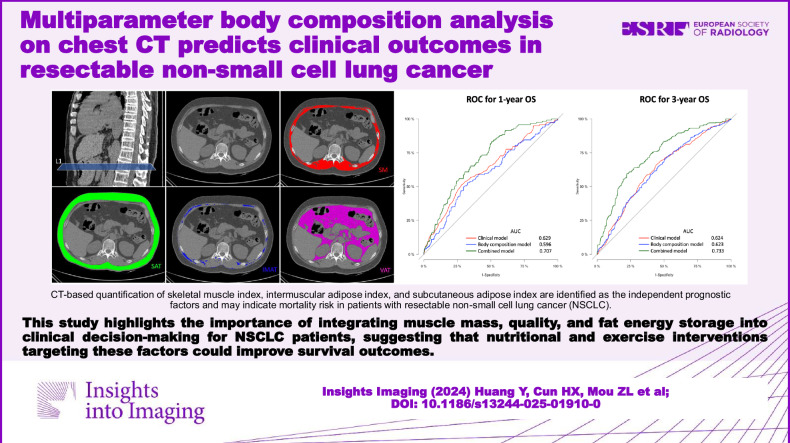

## Introduction

Lung cancer is the leading cause of cancer-related death worldwide, and non‐small‐cell lung cancer (NSCLC) comprises 85% of new cases [1]. Curative resection is the standard treatment for stage I-IIIA NSCLC, offering a survival benefit [2]. Although the tumor node metastasis (TNM) staging system is crucial for prognosis, there remains significant variability in outcomes among patients with the same stage of NSCLC [3, 4]. This highlights the imperative need for understanding other prognostic factors to improve survival in patients with NSCLC.

Body composition substantially affects health outcomes, including cancer prognosis [5, 6]. Low skeletal muscle index (SMI) is associated with a higher risk of tumor-related adverse events, such as surgical complications or poor overall survival (OS) [7, 8]. A higher body mass index (BMI) is a protective factor for lung cancer prognosis, which is a controversial phenomenon known as the *obesity paradox* [9]. Besides, subcutaneous adipose tissue (SAT) appears protective, though visceral adipose tissue (VAT) is associated with increased rectal cancer-related mortality [10]. Although studies have shown that adipose tissue impacts the 5-year OS rate in early-stage NSCLC [11], the relationship between survival in patients with NSCLC and muscle quality and distribution of adipose tissue has not been well-characterized in the literature.

Routine computed tomography (CT) scans can accurately quantify body composition. Body composition at the third lumbar vertebrae level (L3) is currently representative of the whole-body composition [12], but most chest CT scans for NSCLC fail to extend the range to the L3 level. Most recently, several studies have identified that the body composition calculated at the twelfth thoracic vertebra or first lumbar vertebrae (L1) level is accepted and recognized as an alternative level for body composition analysis on chest CT images, as these two levels have strong correlations in the skeletal muscle (SM) area [13, 14]. However, the associations between chest CT-based measurements of body composition at alternative levels and NSCLC outcomes remain uncertain.

Therefore, our study aimed to evaluate the association between baseline chest CT-based metrics of body composition at the L1 level and resectable NSCLC outcomes in a multicenter cohort.

## Materials and methods

### Participants

**Fig. 1:**
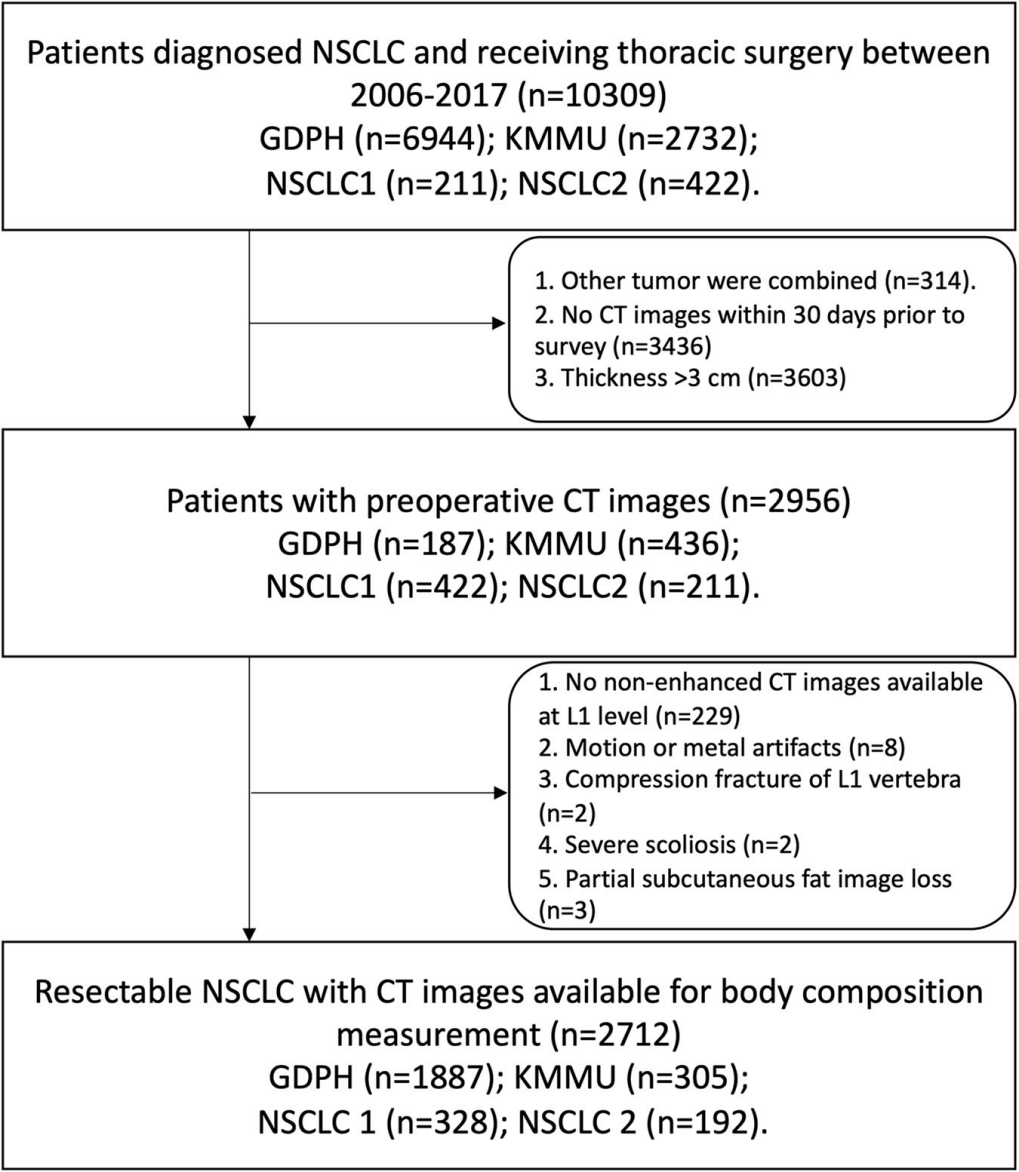
Flowchart shows selected patients diagnosed with NSCLC between 2006 and 2017. NSCLC, non‐small‐cell lung cancer; CT, computed tomography

This retrospective analysis was approved by the institutional review board of Guangdong Provincial People’s Hospital (GDPH) and First Affiliated Hospital of Kunming Medical University (KMMU). The requirement for written informed consent was waived. All data were de-identified and posed no additional risk to the patients. Patients with surgical treatment for NSCLC between January 2006 and December 2017 were retrospectively reviewed in this study from the GDPH and KMMU. And we included two NSCLC CT image data in the TCIA public database (NSCLC1 and NSCLC2) [15, 16]. In the GDPH, KMMU, and NSCLC2 cohorts, 216 patients (9.06%) received chemotherapy. All patients in the GDPH and KMMU cohorts were obtained from China, the NSCLC1 and NSCLC2 were from Americans and Dutch cohorts, respectively. The study flowchart of inclusion and exclusion criteria is presented in Fig. 1.

### Clinical data and patients’ outcomes

We collected clinical data of eligible patients, including gender, age, height, weight, BMI, smoking history, date of surgery, histological subtype, and TNM staging system (8th edition, 2017). Smoking amount was recorded in terms of pack-years (pack-year = numbers of packs of cigarettes/day × years of smoking). All patients were diagnosed with NSCLC by pathology, but the histological subtype was not documented in some cases. In the NSCLC2 database, there were 45 missing TNM stage data points. Follow-up time ended in December 2020 or at an earlier date of death. The primary outcome was OS, defined as the time from the date of first surgery to the date of death. The secondary outcome was disease-free survival (DFS), defined as the time from surgery to any event related to lung cancer (local recurrence, distant metastasis), or death from any cause, whichever occurred first. The date of death and events of tumor recurrence or distant metastasis were determined through telephone follow-up and electronic medical record system searches.

### CT image acquisition

All preoperative chest CT scans were obtained within 1 month before surgery using one of five scanners (Somatom Definition AS or Biograph 40, Siemens; LightSpeed 8 or VCT 64; GE Healthcare; Philips Brilliance iCT 256, Philips Ingenuity) in the GDPH and KMMU cohorts. The scanning parameters are as follows: slice thickness, 1 mm or 3 mm; section spacing, 1 mm or 3 mm; tube voltage, 100 kVp; and tube current, 500 mAs. Two NSCLC data from TCIA did not provide detailed scanning parameters.

### Multiparameter body composition analysis

**Fig. 2:**
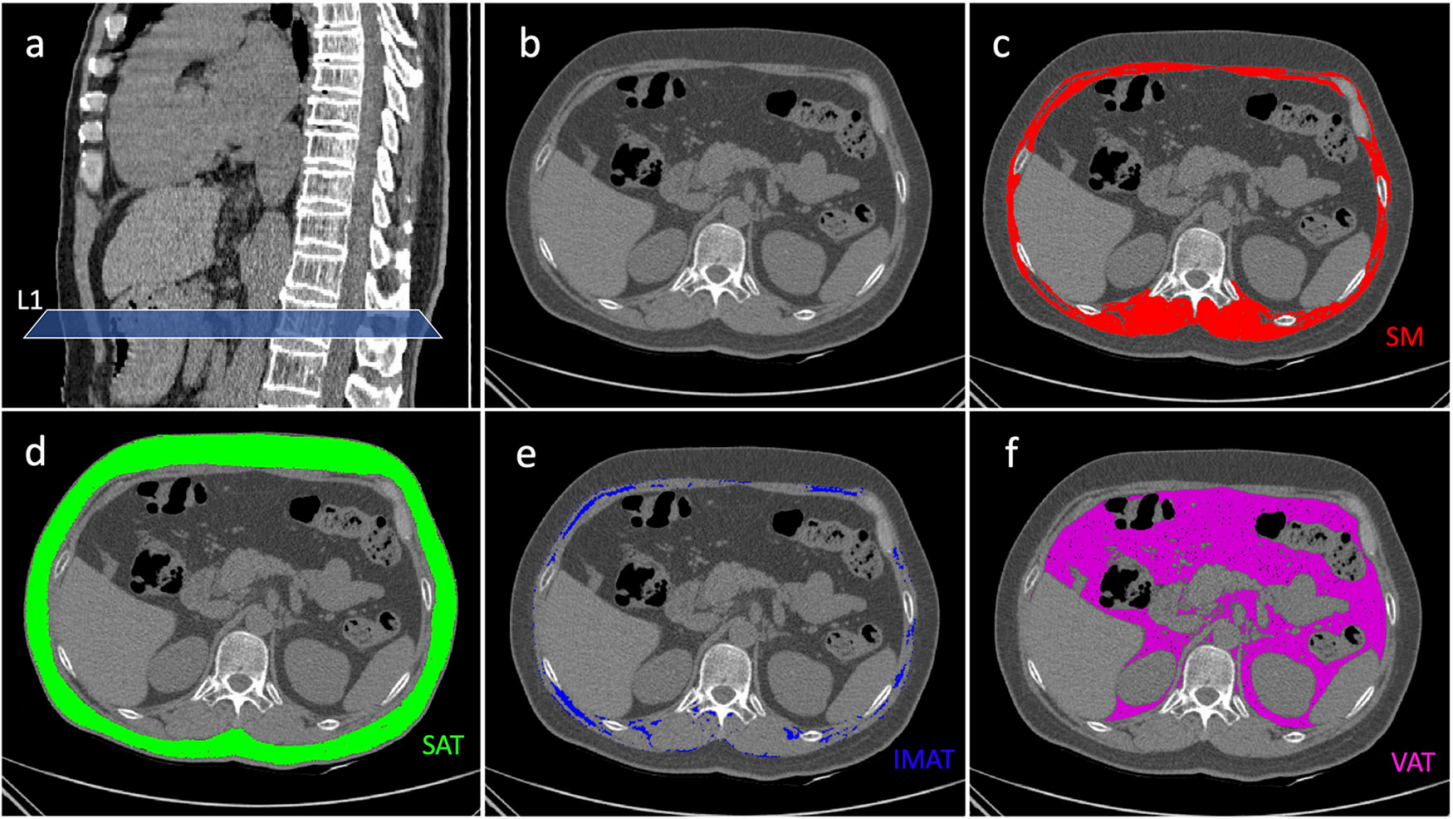
Segmentation of body composition at the level of L1 based on CT image. **a** Body composition segmentation at L1 level. **b** Unsegmented CT image. **c** Segmented skeletal muscle (SM, red). **d** Segmented subcutaneous fat (SAT, green). **e** Segmented intermuscular adipose tissue (IMAT, blue). **f** Segmented visceral adipose tissue (VAT, purple)

For each participant, a single axial non-contrast-enhanced CT image at the middle L1 level was used to semi-automatically segment SM, SAT, VAT, intermuscular adipose tissue (IMAT) by a commercially available software (Slice-O-Matic V5.0, Canada) (Fig. 2). The segmentation was performed by two trained radiology residents (HXC and ZLM), who were blinded to the patient’s clinical data and outcomes. The cases were evenly divided between them. To efficiently assess interobserver agreement, 200 cases were randomly selected for overlapping segmentation. Each measurement was made twice on each patient. CT values ranging from −29 to +150 HU were used to identify muscle, −190 to −30 HU for IMAT and SAT, −150 to −50 HU for VAT.

After completing the four-tissue type segmentation, the areas of SM, SAT, VAT, IMAT, and muscle radiodensity (MD) were calculated as the basic body composition metrics. Then, the skeletal muscle index (SMI), subcutaneous adipose index (SAI), visceral adipose index (VAI), and intramuscular adipose index (IMAI) were calculated by dividing the area of body composition by the square of height in meters to account for body size-related variations in body composition as follows: SMI = SM/*h*^2^; SAI = SAT/*h*^2^; VAI = VAT/*h*^2^; IMAI = IMAT/*h*^2^. To maximize sample size while maintaining reasonable accuracy, we estimated height for cases with missing data by using the anterior-posterior diameter of the T12 vertebra on an axial slice located in the middle of the vertebra [17], as direct height measurements were unavailable for all NSCLC1 and NSCLC2. Additionally, a parameter calculated as SM×MD that considers both muscle amount and quality was the skeletal muscle gauge (SMG) [18]. Finally, metrics about the distributional relationship of body compositions were calculated as follows: fat fraction (FF) = IMAT/SM, visceral adipose tissue-skeletal muscle ratio (VMR) = VAT/SM [19], visceral adipose tissue-subcutaneous adipose tissue ratio (VSR) = VAT/SAT [20].

### Statistical analysis

Continuous variables were reported as mean ± SD, and categorical variables were presented as frequencies with percentages. The normality of continuous variables was assessed by the Shapiro-Wilk test. Continuous variables with normal distribution were analyzed by independent *t*-test, and continuous variables with non-normal distribution were used Mann–Whitney *U-*test. Categorical variables were compared using the chi-square test. To ensure data accuracy and reliability, analyses were restricted to cases with complete data for key variables (TNM stage and BMI) in order to reduce potential bias from variability in multicenter clinical records. A two-way random effect model with measures of absolute agreement was used to calculate intraclass correlation coefficient (ICC) values, with ICC > 0.75 regarded as “excellent”. For survival analysis, univariate, and multivariable Cox proportional hazards regression analyses were performed. Variables with *p* < 0.05 in the multivariable Cox model were considered significant. Backward stepwise regression analysis was undertaken for model building. The Schoenfeld residual test was applied to continuous variables to assess the proportional hazards assumption. Multicollinearity was assessed using the variance inflation factor (VIF). The clinical model included significant clinical risk indicators, the Body composition model included significant body composition indicators, and the Combined model included both clinical risk and body composition indicators. Receiver operating characteristic (ROC) curves and time-dependent area under the curve (AUC) were used to assess the predictive value. The time-dependent AUC analysis and integrated AUC calculation were performed using R package “riskRegression” and “risksetROC”. The cutoff value was determined by the function “surv_cutpoint” of package “survminer”. The Kaplan–Meier curves were plotted and compared using a log-rank test. Statistical analyses and plotting were performed in the R software (version 4.2.2, R Foundation for Statistical Computing). *p* < 0.05 was considered to indicate a statistical difference.

## Results

### Clinical characteristics and multiparameter body composition

A total of 2712 patients with NSCLC (mean age 61.53 ± 10.97 years, range 19–92 years; 1566 males and 1146 females) were included in the analysis. The clinical characteristics and multiparameter body composition of patients are summarized in Table 1. During the postoperative follow-up period, the median OS for all patients was 37.85 months (IQR, 20.10–70.00 months), while the DFS was 29.72 months (IQR, 3.73–40.63 months). For censored survivors, the median OS was 43.50 months (IQR, 23.17–81.83 months), and the median DFS was 30.43 months (IQR, 3.40–44.07 months). As of the last follow-up date, 635 patients have died (23.41%), and 465 patients have experienced local/regional recurrence and/or distant metastasis (19.51%). There was excellent interobserver agreement (ICC = 0.83–0.99) and excellent intraobserver agreement (ICC = 0.98–1.00) between body composition parameters measured by two readers (Table S1).

**Table 1:** Clinical and body composition characteristics

Characteristic	Overall (*n* = 2712)	Center				
		GDPH (*n* = 1887)	KMMU (*n* = 305)	NSCLC1 (*n* = 328)	NSCLC2 (*n* = 192)	*p*-value
Female, *n* (%)	1146 (42.27%)	820 (43.46%)	144 (47.21%)	113 (34.35%)	69 (35.93%)	0.001*
Age (year)	61.53 ± 10.97	59.85 ± 10.60	61.13 ± 10.91	68.35 ± 9.40	67.61 ± 10.20	< 0.001*
Weight (kg)	66.80 ± 29.46	60.22 ± 10.27	60.73 ± 10.02	—	76.59 ± 18.96	< 0.001*
BMI (kg/m^2^)	22.87 ± 3.45	22.63 ± 3.12	22.91 ± 3.25	—	26.01 ± 5.71	< 0.001*
Smoker, *n* (%)	709 (29.74%)	483 (25.60%)	76 (24.91%)	—	150 (78.13%)	< 0.001*
Pack-years	31.83 ± 27.73	33.00 ± 24.37	27.24 ± 24.09	—	28.96 ± 27.92	< 0.001*
TNM stages (*n* = 2667)						< 0.001*
I, *n* (%)	1663 (62.35%)	1304 (69.10%)	225 (73.77%)	77 (23.48%)	57/147 (38.78%)	
II, *n* (%)	381 (14.29%)	253 (13.41%)	37 (12.13%)	35 (10.67%)	56/147 (38.10%)	
III, *n* (%)	523 (19.61%)	234 (12.40%)	43 (14.10%)	216 (65.85%)	30/147 (20.41%)	
IV, *n* (%)	100 (3.75%)	96 (5.09%)	0 (0.00%)	0 (0.00%)	4/147 (2.72%)	
Histological type						< 0.001*
Adenocarcinoma, *n* (%)	1786 (65.86%)	1330 (70.48%)	258 (84.59%)	40 (12.20%)	158 (82.29%)	
Non-Adenocarcinoma, *n* (%)	926 (34.14%)	557 (29.51%)	47 (15.41%)	288 (87.80%)	34 (17.71%)	
MD (cm^2^)	43.31 ± 8.35	44.92 ± 6.90	48.00 ± 7.15	32.32 ± 7.30	38.80 ± 7.62	< 0.001*
SMG (cm^2^)	2986.49 ± 1065.94	3108.19 ± 963.50	3697.36 ± 1093.69	1914.50 ± 724.68	2496.15 ± 996.16	< 0.001*
SMI (cm^2^/m^2^)	25.17 ± 6.13	25.86 ± 5.87	28.70 ± 5.49	19.86 ± 4.31	21.69 ± 5.41	< 0.001*
SAI (cm^2^/m^2^)	27.90 ± 18.57	26.54 ± 17.12	29.72 ± 16.61	27.79 ± 22.53	38.38 ± 23.62	< 0.001*
IMAI (cm^2^/m^2^)	4.31 ± 3.14	4.09 ± 2.54	3.26 ± 2.36	5.88 ± 5.01	5.50 ± 4.17	< 0.001*
VAI (cm^2^/m^2^)	28.57 ± 21.44	25.58 ± 18.42	25.19 ± 17.32	39.53 ± 27.41	44.82 ± 28.93	< 0.001*
FF	0.18 ± 0.16	0.17 ± 0.12	0.12 ± 0.08	0.31 ± 0.29	0.26 ± 0.20	< 0.001*
VMR	1.17 ± 0.92	0.99 ± 0.71	0.86 ± 0.54	1.98 ± 1.37	1.99 ± 1.14	< 0.001*
VSR	1.40 ± 2.99	1.30 ± 1.94	0.96 ± 0.69	2.38 ± 7.11	1.35 ± 0.88	< 0.001*

Continuous variables are presented as mean ± standard deviation. Categorical variables are presented as numbers (frequency)

*BMI* body mass index, *NSCLC* non-small cell lung cancer, *MD* muscle radiodensity, *SMG* skeletal muscle gauge, *SMI* skeletal muscle index, *SAI* subcutaneous adipose index, *IMAI* intramuscular adipose index, *VAI* visceral adipose index, *FF* fat fraction, *VMR* visceral adipose tissue-skeletal muscle ratio, *VSR* visceral adipose tissue-subcutaneous adipose tissue ratio

* *p*  <  0.05

### Univariate analysis of clinical and body composition parameters

Univariate Cox regression analysis showed that clinical factors of age, gender, weight, BMI, smoking status, histological type, TNM stages were associated with OS (all *p* < 0.05). Among body composition metrics, MD, SMG, SMI, SAI, IMAI, FF, VMR, and VSR were also associated with OS (all *p* < 0.05, Table 2). For DFS, gender, smoking status, pack-years, and TNM stages were prognostic factors (all *p* < 0.05, Table 3). And none of any composition metrics were significant associated with DFS (all *p* > 0.05, Table 3).

**Table 2:** Univariate and multivariable Cox analysis for OS

Covariates	OS					
	Univariate analysis			Multivariate analysis		
	HR	95% CI	*p*-value	HR	95% CI	*p*-value
Age	1.05	1.04, 1.06	< 0.001^*^	1.05	1.03, 1.07	< 0.001^*^
Gender (female)	0.57	0.48, 0.67	< 0.001^*^	—	—	—
Weight	1.01	1.00, 1.01	< 0.001^*^	—	—	—
BMI	0.95	0.91, 0.99	0.007^*^	—	—	—
Smoking status (smoker)	2.31	1.87, 2.58	< 0.001^*^	—	—	—
Pack-years, per 10 units	1.02	0.94, 1.08	0.470	—	—	—
Histological type (squamous cell carcinoma)	2.04	1.88, 2.22	< 0.001^*^	—	—	—
TNM stages						
I	1.00					
II	2.69	2.11, 3.45	< 0.001^*^	2.09	1.62, 2.69	< 0.001^*^
III	5.86	4.85, 7.08	< 0.001^*^	3.45	2.77, 4.30	< 0.001^*^
IV	1.56	0.91, 2.69	0.107	1.89	1.09, 3.28	0.023^*^
MD	1.04	1.03, 0.04	< 0.001^*^	—	—	—
SMG, per 100 units	0.95	0.94, 0.96	< 0.001^*^	—	—	—
SMI	0.92	0.91, 0.94	< 0.001^*^	0.86	0.82, 0.90	< 0.001^*^
SAI	0.99	0.98, 0.99	0.001^*^	0.96	0.95, 0.98	0.001^*^
IMAI	1.09	1.05, 1.09	< 0.001^*^	1.49	1.01, 2.19	0.020^*^
VAI	1.01	1.01, 1.02	< 0.001^*^	—	—	—
FF	4.65	3.53, 6.12	< 0.001^*^	—	—	—
VMR	1.54	1.44, 1.65	< 0.001^*^	—	—	—
VSR	1.04	1.03, 1.05	< 0.001^*^	—	—	—

*OS* overall survival, *HR* hazard ratio, *CI* confidence interval, *BMI* body mass index, *MD* muscle radiodensity, *SMG* skeletal muscle gauge, *SMI* skeletal muscle index, *SAI* subcutaneous adipose index, *IMAI* intramuscular adipose index, *VAI* visceral adipose index, *FF* fat fraction, *VMR* visceral adipose tissue-skeletal muscle ratio, *VSR* visceral adipose tissue-subcutaneous adipose tissue ratio

* *p*  <  0.05

**Table 3:** Univariate and multivariable Cox analysis for DFS

Covariates	DFS					
	Univariate analysis			Multivariate analysis		
	HR	95% CI	*p*-*v*alue	HR	95% CI	*p*-value
Age	1.01	1.00, 1.02	0.166	—	—	—
Gender (female)	0.80	0.66, 0.96	0.016^*^	—	—	—
Weight	0.99	0.97, 1.01	0.808	—	—	—
BMI	1.01	0.96, 1.06	0.104	—	—	—
Smoking status (smoker)	1.28	1.06, 1.54	0.012^*^	—	—	—
Pack-years, per 10 units	1.07	1.01, 1.12	0.011^*^	1.07	1.01, 1.13	0.039^*^
Histological type (squamous cell carcinoma)	1.23	0.89, 1.7	0.323	—	—	—
TNM stages						
I	1.00					
II	2.29	1.75, 3.00	< 0.001^*^	2.28	1.75, 2.99	< 0.001^*^
III	3.89	3.10, 5.00	< 0.001^*^	3.77	3.00, 4.80	< 0.001^*^
IV	7.15	5.00, 10.2	< 0.001^*^	7.19	5.02, 10.30	< 0.001^*^
MD	1.02	1.00, 1.04	0.339	—	—	—
SMG, per 100 units	1.00	0.99, 1.01	0.475	—	—	—
SMI	1.00	0.98, 1.03	0.273	—	—	—
SAI	0.99	0.98, 1.00	0.232	—	—	—
IMAI	1.03	0.99, 1.08	0.764	—	—	—
VAI	1.99	0.99, 1.01	0.138	—	—	—
FF	0.93	0.85, 1.08	0.856	—	—	—
VMR	0.96	0.79, 1.17	0.485	—	—	—
VSR	1.03	0.99, 1.09	0.198	—	—	—

*DFS* disease-free survival, *HR* hazard ratio, *CI* confidence interval, *BMI* body mass index, *MD* muscle radiodensity, *SMG* skeletal muscle gauge, *SMI* skeletal muscle index, *SAI* subcutaneous adipose index, *IMAI* intramuscular adipose index, *VAI* visceral adipose index, *FF* fat fraction, *VMR* visceral adipose tissue-skeletal muscle ratio, *VSR* visceral adipose tissue-subcutaneous adipose tissue ratio

* *p*  <  0.05

### Multivariable analysis of clinical and body composition parameters

**Fig. 3:**
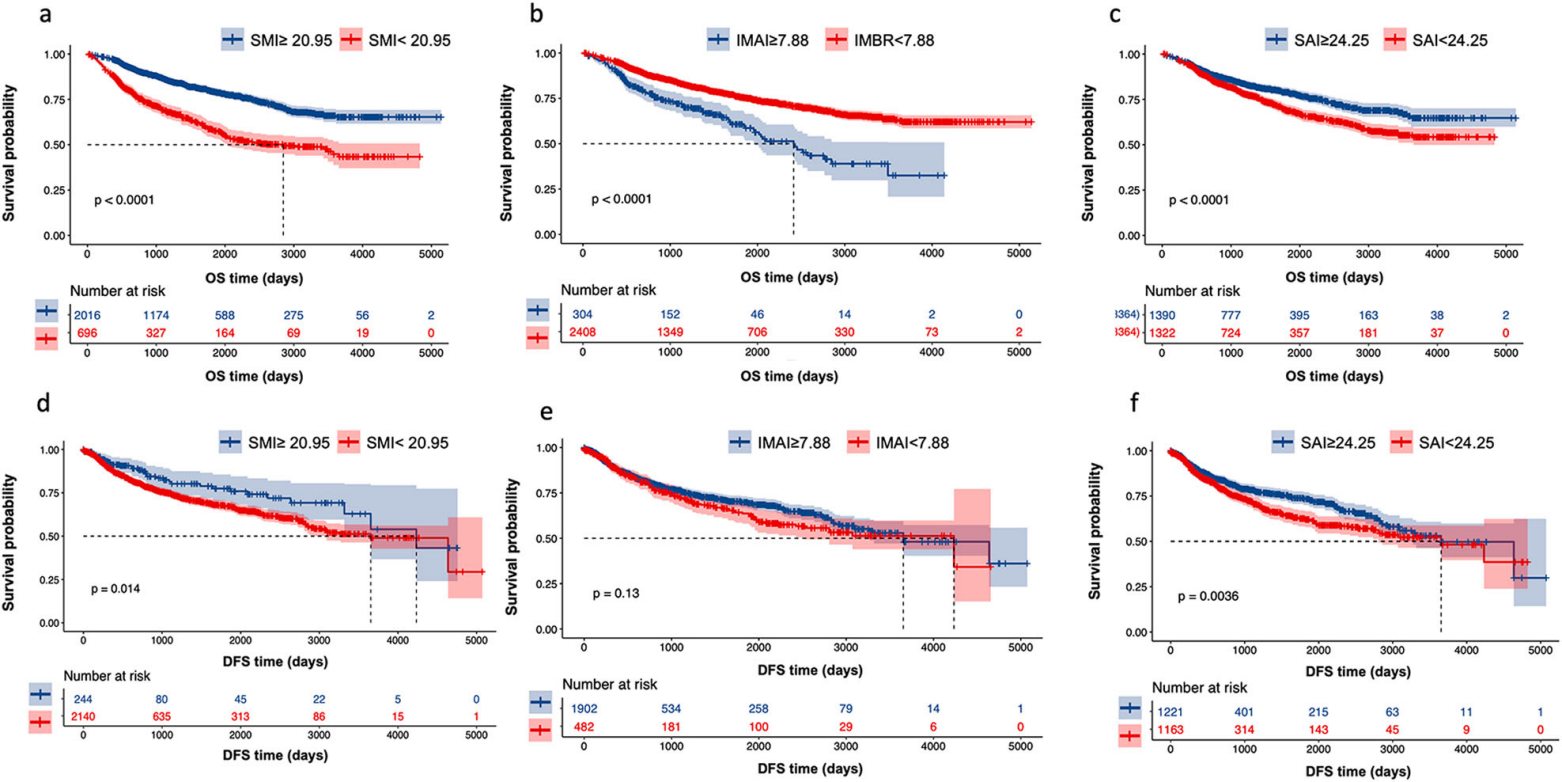
Kaplan–Meier survival curves showing the overall survival according and disease-free survival to SMI, IMAI, and SAI. **a**, **d** SMI in the overall cohort. **b**, **e** IMAI in the overall cohort. **c**, **f** SAI in the overall cohort. SMI, skeletal muscle index; IMAI, intermuscular adipose tissue index; SAI, subcutaneous adipose index

**Fig. 4:**
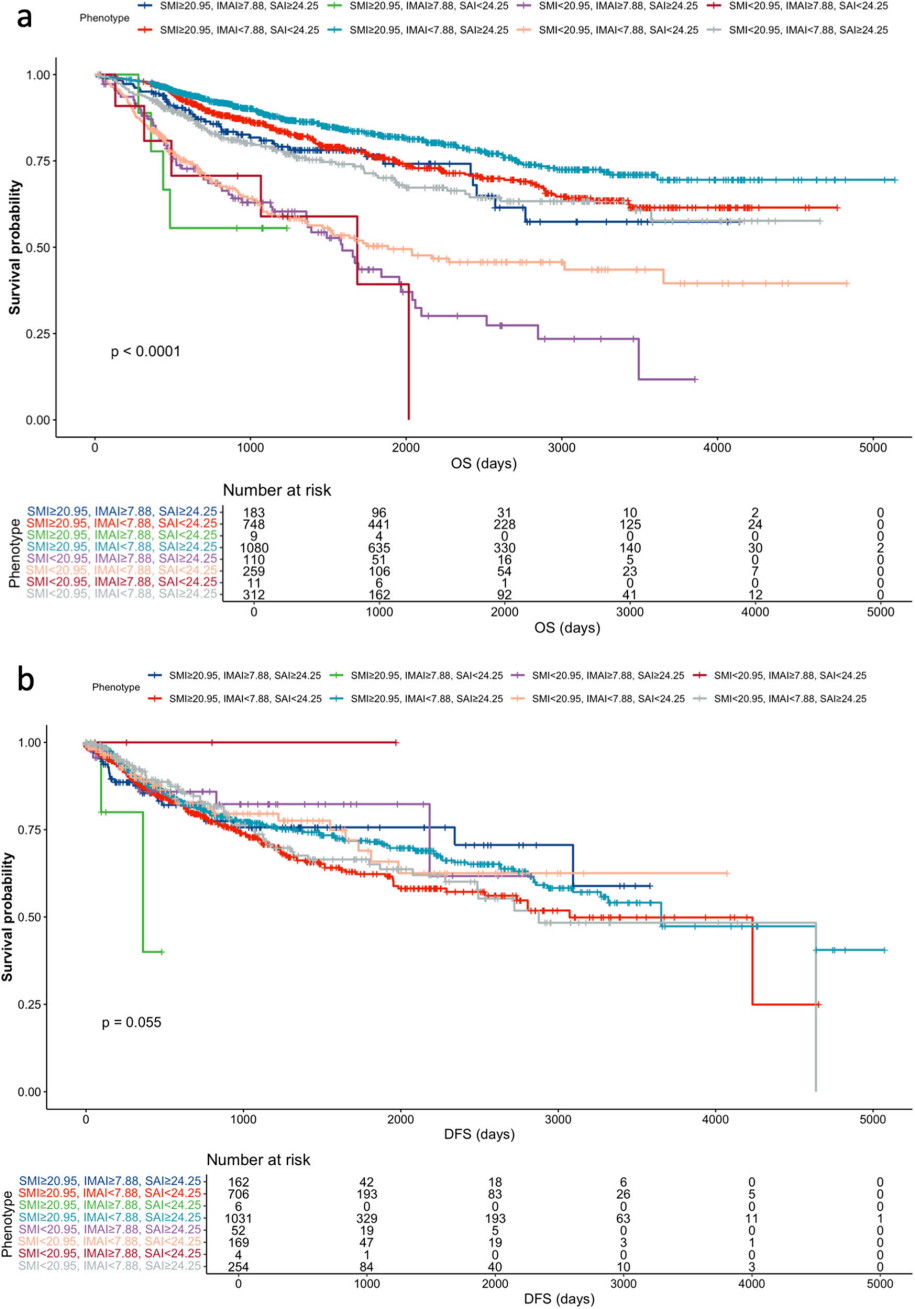
Kaplan–Meier curves show overall survival and disease-free survival according to comprehensive body composition phenotypes. **a** Overall survival. **b** Disease-free survival. OS, overall survival; AUC, area under the curve; DFS, disease-free survival; SMI, skeletal muscle index; IMAI, intermuscular adipose tissue index; SAI, subcutaneous adipose index

In the multivariable analysis, age (HR = 1.05; 95% CI: 1.03, 1.07; *p* < 0.001), TNM stages (II stage HR = 2.09; 95% CI: 1.62, 2.69; *p* < 0.001; III stage HR = 3.45; 95% CI: 2.77, 4.30; *p* < 0.001, IV stage HR = 1.89; 95% CI: 1.09, 3.28; *p* < 0.023), SMI (HR = 0.86; 95% CI: 0.82, 0.90; *p* < 0.001), SAI (HR = 0.96; 95% CI: 0.95, 0.98; *p* = 0.001), and IMAI (HR = 1.49; 95% CI: 1.01, 2.19; (*p* = 0.020) were the independent factors for OS (Table 2). In the body composition parameters, SMI, SAI, and IMAI were strongly prognostic in the log-rank test and represented the prognostic value for OS (Table 2 and Fig. 3). The Schoenfeld residual plot did not provide any indication that the proportional hazards assumption was violated (Fig. S1). Figure 4 shows a Kaplan–Meier curve according to the body composition phenotypes of SMI, IMAI, and SAI categories. Patients with high SMI, SAI, and low IMAI, and showed the highest survival rate. For DFS, TNM stage (II stage HR = 2.28; 95% CI: 1.75, 2.99; *p* < 0.001; III stage HR = 3.77; 95% CI: 3.00, 4.80; *p* < 0.001, IV stage HR = 7.19; 95% CI: 5.02, 10.3; *p* < 0.001), and pack-years (HR = 1.07; 95% CI: 1.01, 1.13; *p* < 0.039) were associated with DFS (Table 3). All body composition parameters were not statistically associated with DFS (*p* > 0.05). In the Cox regression multivariable analyses, BMI was not prognostic for OS or DFS (all *p* > 0.05, Tables 2 and 3).

### Analyses stratified by gender, tumor stage, and center cohort

Due to the potential influence of differences in body composition across gender, tumor stage, and center on the results. The results of the stratified analyses by gender, tumor stages, and center cohort are provided in Supplementary Table S2 and Figs. S2–4. Body composition parameters of IMAI and SAI were still significantly associated with OS in the male or female group (*p* < 0.05, Fig. S2). And similar results were found in each TNM stage, and center (Figs. S3–4).

### Predictive ability of body composition parameters

**Fig. 5:**
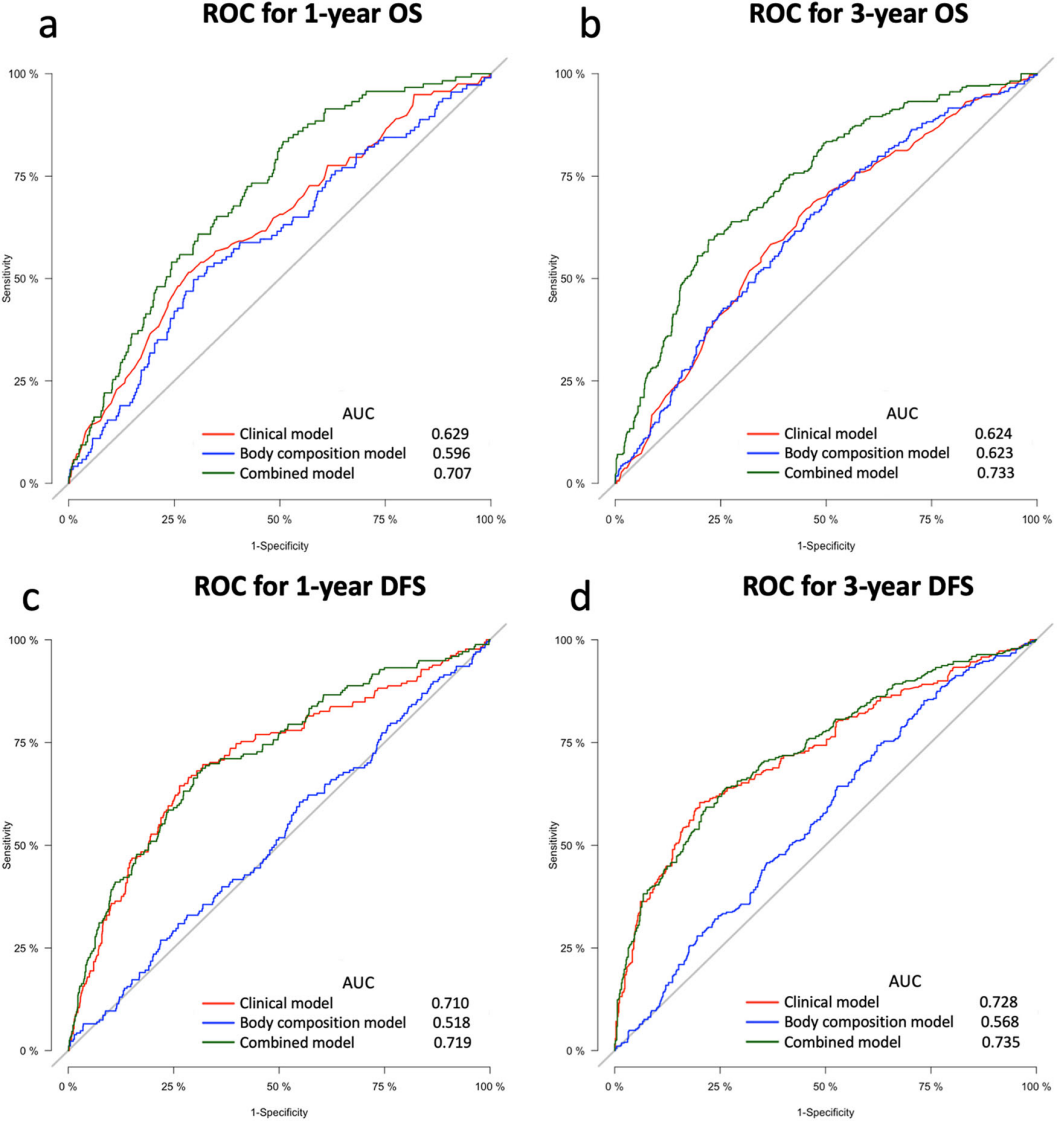
Receiver operating characteristic curves for Cox regression model in overall survival and disease-free survival analysis. **a** 1-year OS prediction. **b** 3-year OS prediction. **c** 1-year DFS prediction. **d** 3-year DFS prediction. Age and TNM stages are included in the clinical model for predicting OS. Pack-years and TNM stages are included in the clinical model for predicting DFS. The body composition model includes skeletal muscle index, subcutaneous adipose index, and intermuscular adipose tissue index. The combined model includes all the factors in both the clinical model and the body composition model. ROC, receiver operating characteristic curve; OS, overall survival; AUC, area under the curve; DFS, disease-free survival

We independently validated the predictive ability of three Cox regression models, including the clinical model, body composition model, and combined model (Fig. 5). The result of VIF indicated that multicollinearity was not a concern (age, VIF  =  1.07; TNM stages, VIF  =  1.046; SMI, VIF  = 1.06; IMAI, VIF  = 1.75; SAI, VIF  = 1.68). In patients who completed 1-year and 3-year follow-ups, the inclusion of body composition parameters significantly improved the AUC of the clinical model for overall survival, increasing from 0.629 and 0.627 to 0.707 and 0.733, respectively (all *p * < 0.05). However, in predicting 1-year and 3-year DFS, body composition parameters did not provide significant performance enhancement for the clinical model. Time-dependent AUC analysis showed that the combined model had the best performance in predicting OS (Fig. S5). Moreover, the integrated AUC for the combined model (0.726) was significantly higher than that of the clinical model (0.628) and the body composition model (0.609) (all *p* < 0.05).

## Discussion

Body composition is a well-known marker for the prognosis of patients with cancer. In our comprehensive analysis of body composition, we found the prognostic value of SMI, IMAI, and SAI calculated from preoperative chest CT in patients with NSCLC. We first demonstrated here that high IMAI and SAI based on baseline chest CT were independently associated with OS and rather than BMI and VAT for in patients with NSCLC. Our results are robust in a series of stratified analyses by gender, TNM stages, and centers.

Most research on body composition and cancer outcomes has focused on SMI. Recently, low muscle mass has been linked to poorer survival in cancer patients [21, 22]. Our study also demonstrates a significant association between muscle mass and clinical outcomes in patients with NSCLC, consistent with previous findings, even when SM measurements are performed on chest CT. Muscle mass is an essential source of amino acids and energy, crucial for maintaining the body’s protein balance and meeting its metabolic demands during illness. It also plays a vital role in sustaining immune function. A reduction in muscle mass can weaken the immune system, making it more challenging for the body to fight cancer and recover from treatment [23]. NSCLC patients with greater muscle mass typically tolerate surgical treatments better, experiencing fewer and less severe side effects. Muscle mass also helps mitigate the catabolic effects of treatments, promoting faster recovery and reducing complications [24].

Interestingly, our results found that SM quality was associated with survival outcomes, as confirmed by more muscle fat infiltration in patients with NSCLC with poor clinical outcomes. Nowadays, MD and IMAT are the most used metrics for quantizing muscle fat infiltration [25, 26]. For MD, although a previous study demonstrated that NSCLC patients with lower paravertebral MD had a poor survival prognosis [27], we did not find a statistical association between MD and clinical outcomes. This may be due to the fact that MD is highly dependent on CT instrumentation [28], which might limit its generalization. Additionally, MD measurement dependence on segmentation thresholds of SM may have led to an underestimation of intramuscular fat, which could explain the reduced association in the adjusted model. Furthermore, IMAI instead of IMAT was independently associated with OS in patients with NSCLC in our study, and our findings also underscore the prognostic value of IMAI in additional analysis of stratified by gender, tumor stage, and center. The finding suggested the body composition parameters used in clinical practice should be standardized to balance individual differences. Compared to previous metrics, IMAI provides a more accurate measure of intramuscular fat. Additionally, the exact biological mechanism by which muscle fat infiltration affects survival remains incompletely understood. Nevertheless, in a previous resected colorectal cancer study, the reduced survival rate in patients with low muscle quality could result from high neutrophil-to-lymphocyte ratios, markers of systemic inflammation, and metabolic dysfunction [29].

BMI does not distinguish between SM and fat, which has led to the interpretation of the improvement in OS in obese lung cancer patients as “obesity paradox”. Notably, our research indicates that SAI plays a primary role in the obesity paradox, rather than VAT-related parameters. This might be due to SAI providing energy reserves and tumor‐suppressing effects in patients with metabolically healthy obesity [30]. A previous study has also found the protective effect of SAT on the prognosis of gastric cancer, as well as the detrimental effect of VAT on its outcomes [31]. However, our result showed that abdominal VAT is not a significant negative factor for survival in NSCLC. This result implies that abdominal VAT may not be suitable for prognosis prediction in NSCLC. However, mediastinal VAT may provide potential prognostic value for patients with NSCLC, which warrants further investigation.

A previous study has found high SAT was correlated with better DFS in patients with colorectal cancer [32]. However, the role of body composition may vary across different types of cancer. Consistent with other lung cancer studies, we observed no significant independent effect of any body composition parameters on DFS in patients with NSCLC [11, 25]. In this study, there was a differential impact of body composition on OS and DFS in patients with NSCLC. This could suggest that body composition parameters do not reflect the aggressiveness of NSCLC but act mainly as an indicator of the overall fragility of the host [26].

This study has several limitations. First, this retrospective study spans a long period, during which treatment approaches, CT technology, and other factors may have evolved. And patients had missing information on BMI (12.09%) in the NSCLC1 center and TNM stage (1.65%) in the NSCLC2 center. The height was not available for patients in the NSCLC1 and NSCLC2; thus, we had to estimate the height by using the anteroposterior diameter of the T12 vertebra, an approach that has been previously validated [17]. Second, we applied a consistent cutoff across all subgroup analyses rather than calculating optimal thresholds for each subgroup. While this avoids data-specific optimization and allows for testing the general applicability of body composition as a prognostic indicator, it may reduce sensitivity within specific subgroups. Third, manual body composition segmentation using high-resolution CT images is a labor-intensive and time-consuming process. Differences may exist between single-slice and whole-body body composition measurements. Fourth, there may be additional prognostic factors not addressed in this study, such as nutritional status, performance status, muscle strength, and respiratory function.

## Conclusion

In conclusion, our study showed the potential clinical value of SMI, IMAI, and SAI as independent risk factors for OS of resectable NSCLC patients. By integrating multiparametric body composition analysis with traditional risk factors, high-risk patients with poor prognosis can be identified early, enabling timely intervention. Although clinical information continues to be significant, our study emphasizes the significance of muscle quality measures and energy storage in clinical decision-making for patients with NSCLC. Reversing negative muscle quality through targeted nutritional and exercise support for patients may represent a potential strategy for improving survival outcomes in at-risk patients with NSCLC. Future prospective studies are needed to validate these findings and establish the clinical utility of body composition parameters in routine practice.

## Supplementary information

ELECTRONIC SUPPLEMENTARY MATERIAL

## Data Availability

The datasets generated or analyzed during the study are available from the corresponding author upon reasonable request.

## Abbreviations

AUC: Area under the curve
BMI: Body mass index
CT: Computed tomography
DFS: Disease-free survival
FF: Fat fraction
ICC: Intraclass correlation coefficient
IMAT: Intermuscular adipose tissue
L1: First lumbar vertebrae
L3: Third lumbar vertebrae
MD: Muscle radiodensity
NSCLC: Non-small-cell lung cancer
OS: Overall survival
SAI: Subcutaneous adipose index
SAT: Subcutaneous adipose tissue
SM: Skeletal muscle
SMI: Skeletal muscle index
TNM: Tumor node metastasis
VAT: Visceral adipose tissue
VIF: Variance inflation factor
VMR: Visceral adipose tissue-skeletal muscle ratio
VSR: Visceral adipose tissue-subcutaneous adipose tissue ratio

## References

[1] Sung H, Ferlay J, Siegel RL et al (2021) Global Cancer Statistics 2020: GLOBOCAN estimates of incidence and mortality worldwide for 36 cancers in 185 countries. CA Cancer J Clin 71:209–249 33538338 10.3322/caac.21660

[2] de Koning HJ, van der Aalst CM, de Jong PA et al (2020) Reduced lung-cancer mortality with volume CT screening in a randomized trial. N Engl J Med 382:503–513 31995683 10.1056/NEJMoa1911793

[3] Kratz JR, Haro GJ, Cook NR et al (2019) Incorporation of a molecular prognostic classifier improves conventional non-small cell lung cancer staging. J Thorac Oncol 14:1223–1232 30959120 10.1016/j.jtho.2019.03.015

[4] Huang Y, Liu Z, He L et al (2016) Radiomics signature: a potential biomarker for the prediction of disease-free survival in early-stage (I or II) non-small cell lung cancer. Radiology 281:947–957 27347764 10.1148/radiol.2016152234

[5] Al-Sawaf O, Weiss J, Skrzypski M et al (2023) Body composition and lung cancer-associated cachexia in TRACERx. Nat Med 29:846–858 37045997 10.1038/s41591-023-02232-8 PMC7614477

[6] Wang J, Tan S, Gianotti L, Wu G (2023) Evaluation and management of body composition changes in cancer patients. Nutrition 114:112132 37441827 10.1016/j.nut.2023.112132

[7] Vedire Y, Nitsche L, Tiadjeri M et al (2023) Skeletal muscle index is associated with long term outcomes after lobectomy for non-small cell lung cancer. BMC Cancer 23:778 37598139 10.1186/s12885-023-11210-9 PMC10439565

[8] Yang J, Chen K, Zheng C et al (2022) Impact of sarcopenia on outcomes of patients undergoing liver resection for hepatocellular carcinoma. J Cachexia Sarcopenia Muscle 13:2383–2392 35854105 10.1002/jcsm.13040 PMC9530540

[9] Petrelli F, Cortellini A, Indini A et al (2021) Association of obesity with survival outcomes in patients with cancer: a systematic review and meta-analysis. JAMA Netw Open 4:e213520 33779745 10.1001/jamanetworkopen.2021.3520 PMC8008284

[10] Pellegrini M, Besutti G, Ottone M et al (2023) Abdominal fat characteristics and mortality in rectal cancer: a retrospective study. Nutrients 15:374 10.3390/nu15020374 PMC9864407 36678245

[11] Choi H, Park YS, Na KJ et al (2021) Association of adipopenia at preoperative PET/CT with mortality in stage I non-small cell lung cancer. Radiology 301:645–653 34609197 10.1148/radiol.2021210576

[12] Prado CM, Lieffers JR, McCargar LJ et al (2008) Prevalence and clinical implications of sarcopenic obesity in patients with solid tumours of the respiratory and gastrointestinal tracts: a population-based study. Lancet Oncol 9:629–635 18539529 10.1016/S1470-2045(08)70153-0

[13] Pickhardt PJ (2022) Value-added opportunistic CT screening: state of the art. Radiology 303:241–254 35289661 10.1148/radiol.211561 PMC9083232

[14] Shen Y, Luo L, Fu H et al (2022) Chest computed tomography-derived muscle mass and quality indicators, in-hospital outcomes, and costs in older inpatients. J Cachexia Sarcopenia Muscle 13:966–975 35178898 10.1002/jcsm.12948 PMC8977961

[15] Bakr S, Gevaert O, Echegaray S et al (2018) A radiogenomic dataset of non-small cell lung cancer. Sci Data 5:180202 30325352 10.1038/sdata.2018.202 PMC6190740

[16] Aerts HJ, Velazquez ER, Leijenaar RT et al (2014) Decoding tumour phenotype by noninvasive imaging using a quantitative radiomics approach. Nat Commun 5:4006 24892406 10.1038/ncomms5006 PMC4059926

[17] Waduud MA, Sucharitkul PPJ, Drozd M, Gupta A, Hammond C, Ashbridge Scott DJ (2019) Validation of two-dimensional vertebral body parameters in estimating patient height in elderly patients. Br J Radiol 92:20190342 31596119 10.1259/bjr.20190342 PMC6913349

[18] Marquardt JP, Roeland EJ, Van Seventer EE et al (2022) Percentile-based averaging and skeletal muscle gauge improve body composition analysis: validation at multiple vertebral levels. J Cachexia Sarcopenia Muscle 13:190–202 34729952 10.1002/jcsm.12848 PMC8818648

[19] de Leeuw SP, Pruis MA, Sikkema BJ et al (2023) Analysis of serious weight gain in patients using alectinib for ALK-positive lung cancer. J Thorac Oncol 18:1017–1030 37001858 10.1016/j.jtho.2023.03.020

[20] Pickhardt PJ, Graffy PM, Zea R et al (2020) Automated abdominal CT imaging biomarkers for opportunistic prediction of future major osteoporotic fractures in asymptomatic adults. Radiology 297:64–72 32780005 10.1148/radiol.2020200466 PMC7526945

[21] Suzuki Y, Okamoto T, Fujishita T et al (2016) Clinical implications of sarcopenia in patients undergoing complete resection for early non-small cell lung cancer. Lung Cancer 101:92–97 27794415 10.1016/j.lungcan.2016.08.007

[22] Kim EY, Kim YS, Park I, Ahn HK, Cho EK, Jeong YM (2015) Prognostic significance of CT-determined sarcopenia in patients with small-cell lung cancer. J Thorac Oncol 10:1795–1799 26484630 10.1097/JTO.0000000000000690

[23] Yip C, Dinkel C, Mahajan A, Siddique M, Cook GJ, Goh V (2015) Imaging body composition in cancer patients: visceral obesity, sarcopenia and sarcopenic obesity may impact on clinical outcome. Insights Imaging 6:489–497 26070723 10.1007/s13244-015-0414-0 PMC4519815

[24] Nakamura R, Inage Y, Tobita R et al (2018) Sarcopenia in resected NSCLC: effect on postoperative outcomes. J Thorac Oncol 13:895–903 29751134 10.1016/j.jtho.2018.04.035

[25] Feng S, Mu H, Hou R et al (2022) Prognostic value of myosteatosis in patients with lung cancer: a systematic review and meta-analysis. Int J Clin Oncol 27:1127–1138 35604501 10.1007/s10147-022-02181-1

[26] Lee CM, Kang J (2020) Prognostic impact of myosteatosis in patients with colorectal cancer: a systematic review and meta-analysis. J Cachexia Sarcopenia Muscle 11:1270–1282 32483936 10.1002/jcsm.12575 PMC7567135

[27] Çınar HU, Çelik B, Taşkın G, İnce Ö (2022) Impact of preoperative computed tomography-determined quantity and quality of skeletal muscle on survival after resected non-small cell lung carcinoma. Eur J Surg Oncol 48:1937–1946 35361518 10.1016/j.ejso.2022.03.009

[28] Pigneur F, Luciani A, Ghosn M, Reizine E, Morel A, Stehlé T (2023) Skeletal muscle density is highly dependent on CT instrumentation. Radiology. 10.1148/radiol.222839:222839 10.1148/radiol.222839 37158723

[29] Malietzis G, Johns N, Al-Hassi HO et al (2016) Low muscularity and myosteatosis is related to the host systemic inflammatory response in patients undergoing surgery for colorectal cancer. Ann Surg 263:320–325 25643288 10.1097/SLA.0000000000001113

[30] Lee JH, Yoon YC, Kim HS et al (2022) Obesity is associated with improved postoperative overall survival, independent of skeletal muscle mass in lung adenocarcinoma. J Cachexia Sarcopenia Muscle 13:1076–1086 35212195 10.1002/jcsm.12956 PMC8978026

[31] Han J, Tang M, Lu C, Shen L, She J, Wu G (2021) Subcutaneous, but not visceral, adipose tissue as a marker for prognosis in gastric cancer patients with cachexia. Clin Nutr 40:5156–5161 34461589 10.1016/j.clnu.2021.08.003

[32] Kim JM, Chung E, Cho ES et al (2021) Impact of subcutaneous and visceral fat adiposity in patients with colorectal cancer. Clin Nutr 40:5631–5638 34662848 10.1016/j.clnu.2021.10.001

