# Supplementary material for: Multiparameter body composition analysis on chest CT predicts clinical outcomes in resectable non-small cell lung cancer
Source: Insights Imaging. 2025 Feb 6;16:32. doi: 10.1186/s13244-025-01910-0 (PMC11803022; doi:10.1186/s13244-025-01910-0)
Supplement: Supplementary file 1 — ELECTRONIC SUPPLEMENTARY MATERIAL [file 13244_2025_1910_MOESM1_ESM.pdf]

Multiparameter body composition analysis on chest CT predicts clinical outcomes in resectable non-small cell lung cancer

ELECTRONIC SUPPLEMENTARY MATERIAL

Captions for supplementary material

Supplementary Table S1. Intra-reviewer reliability for the body composition measurements

Supplementary Table S2. Multiparameter body composition by gender and TNM stages

Supplementary Figure S1. Kaplan–Meier survival curves showing the overall survival according to gender.

Supplementary Figure S2. Kaplan–M0eier survival curves showing the overall survival according to TNM stage.

Supplementary Figure S3. Kaplan–Meier survival curves showing the overall survival according to four centers.

Supplementary Table S1. Intra-reviewer reliability for the body composition measurements

Body composition measurements	Interobserver agreement			Intraobserver agreement		
	ICC	95% CI	p	ICC	95% CI	p
SM	0.81	0.70, 0.92	<0.001	0.99	0.97，1.00	<0.001
SAT	0.98	0.92,0.99	<0.001	0.99	0.99，1.00	<0.001
IMAT	0.84	0.79,0.88	<0.001	0.99	0.98，1.00	<0.001
VAT	0.97	0.96,0.98	<0.001	0.99	0.99，1.000	<0.001
MD	0.77	0.67,0.84	<0.001	0.99	0.97，1.00	<0.001

Note. ICC = intraclass correlation coefficient, SM = skeletal muscle, SAT = subcutaneous adipose tissue, IMAT = intermuscular adipose tissue, VAT = visceral adipose tissue, MD = muscle radiodensity.

Supplementary Table S2 Multiparameter body composition by gender and TNM stages

Body Composition parameter	Gender			TNM stage				
	Male	Female	P Value	I stage	II stage	III stage	IV stage	P Value
SM (cm^2)	76.76±17.26	56.47±12.16	＜0.001*	68.26±18.39	70.53±18.31	65.90±17.42	70.69±20.44	0.001*
SAT (cm^2)	57.46±36.06	96.37±50.97	＜0.001*	74.95±45.55	71.55±48.98	73.41±50.20	63.67±40.17	0.093
IMAT (cm^2)	11.52±8.76	11.92±8.61	0.238	11.22±8.26	12.31±8.89	12.61±9.92	10.86±7.23	0.005*
VAT (cm^2)	94.95±70.44	56.73±40.24	＜0.001*	74.73±59.84	85.87±64.36	89.97±72.16	65.70±49.45	0.000*
MD (cm^2)	44.24±8.18	42.04±8.41	＜0.001*	44.12±7.89	42.67±8.46	40.59±9.41	45.61±6.95	0.000*
SMG (cm^2)	3428.70±1070.27	2381.68±700.56	＜0.001*	3036.16±1044.32	3055.13±1092.29	2728.80±1096.68	3220.95±1030.44	0.000*
SMI (cm^2/m^2)	28.33±5.87	23.63±4.79	＜0.001*	26.05±6.02	27.14±5.87	26.24±5.26	26.09±6.46	0.045*
SAI (cm^2/m^2)	18.29±10.08	37.99±17.79	＜0.001*	28.23±16.98	24.78±18.57	25.40±17.51	23.98±15.97	0.001*
IMAI (cm^2/m^2)	3.57±2.33	4.49±2.68	＜0.001*	3.98±2.50	4.12±2.88	3.73±2.45	4.11±2.78	0.318
VAI (cm^2/m^2)	28.13±19.76	22.22±15.58	＜0.001*	25.74±18.73	26.01±17.18	24.54±17.62	24.18±17.78	0.649
FF	0.16±0.14	0.22±0.18	＜0.001*	0.18±0.16	0.19±0.16	0.21±0.18	0.16±0.11	0.001*
VMR	1.28±1.01	1.02±0.75	＜0.001*	1.10±0.87	1.24±0.94	1.39±1.08	0.92±0.64	0.000*
VSR	1.99±3.82	0.59±0.35	＜0.001*	1.25±1.78	1.66±2.91	1.76±5.58	1.39±1.80	0.005*

Note. Data are mean ± SD. SM = skeletal muscle, SAT = subcutaneous adipose tissue, IMAT = intermuscular adipose tissue, VAT = visceral adipose tissue, MD = muscle radiodensity, SMG = skeletal muscle gauge, SMI = skeletal muscle index, SAI = subcutaneous adipose index, IMAI = intramuscular adipose index, VAI = visceral adipose index, FF = fat fraction, VMR = visceral adipose tissue-skeletal muscle ratio, VSR = visceral adipose tissue-subcutaneous adipose tissue ratio. * $p<0.05$.

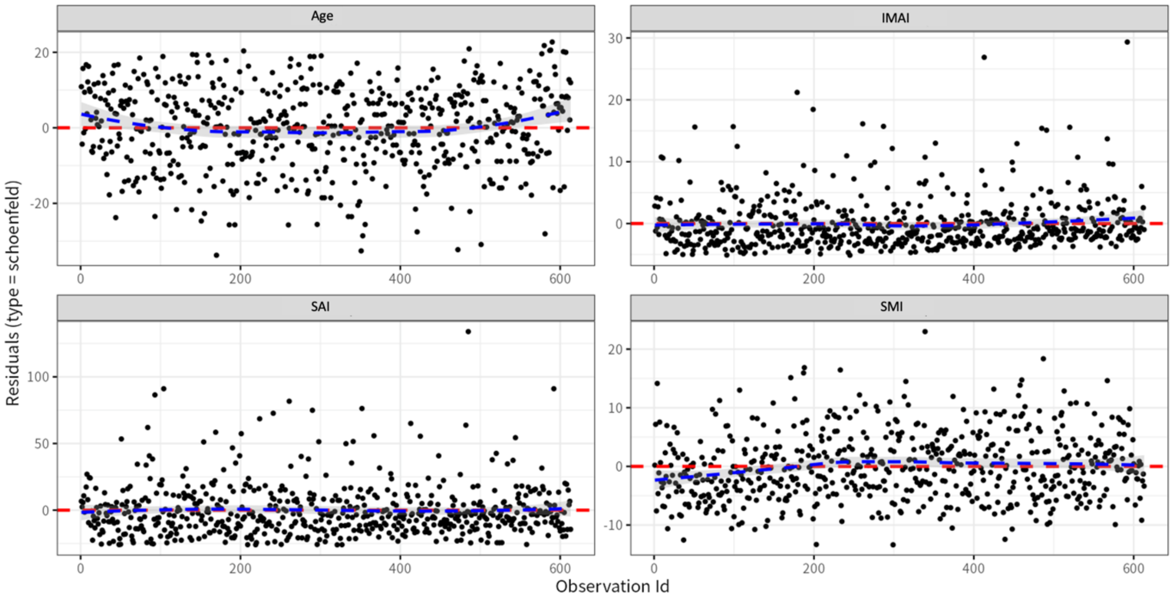

Supplementary Fig. S1 The Schoenfeld residual plot.

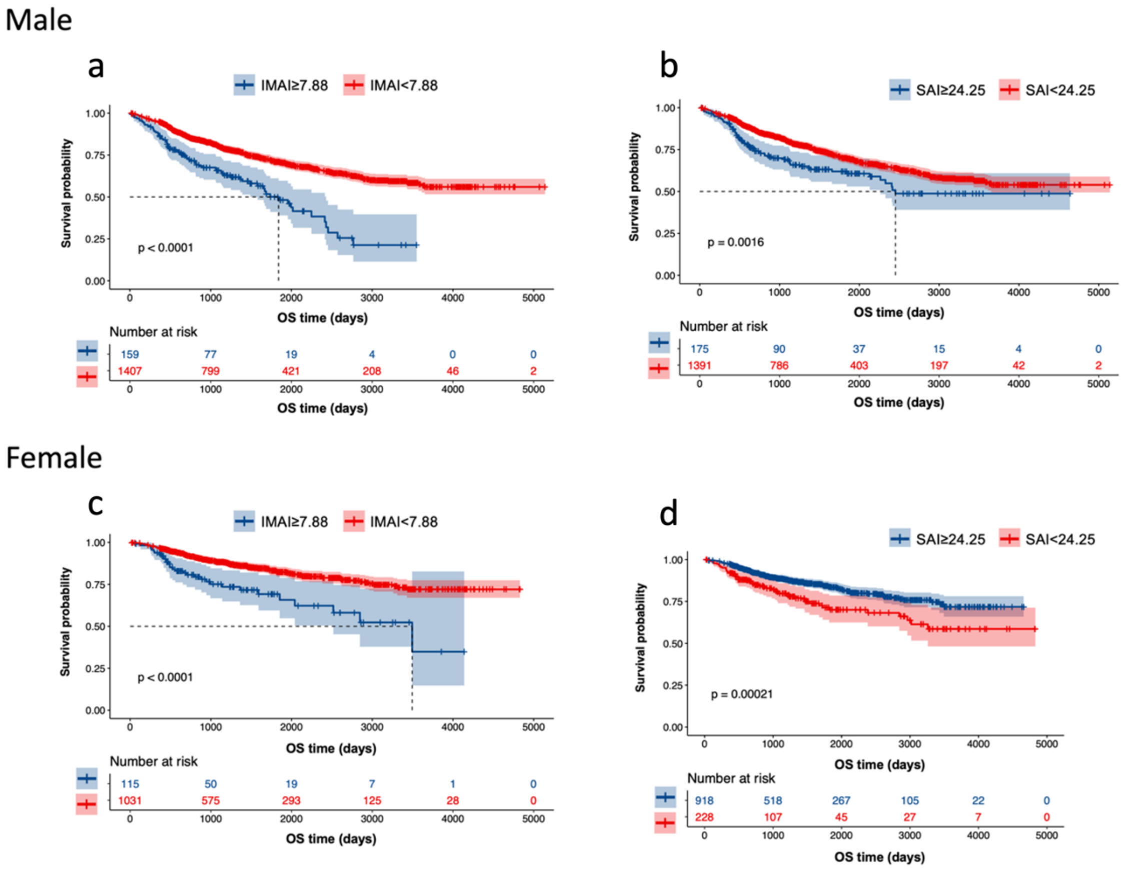

Supplementary Fig. S2 Kaplan–Meier survival curves showing the overall survival according to gender. a, b IMAI and SAI in the male cohort. **c, d** IMAI and SAI in the female cohort. OS = overall survival, IMAI = intramuscular adipose index, SAI = subcutaneous adipose index.

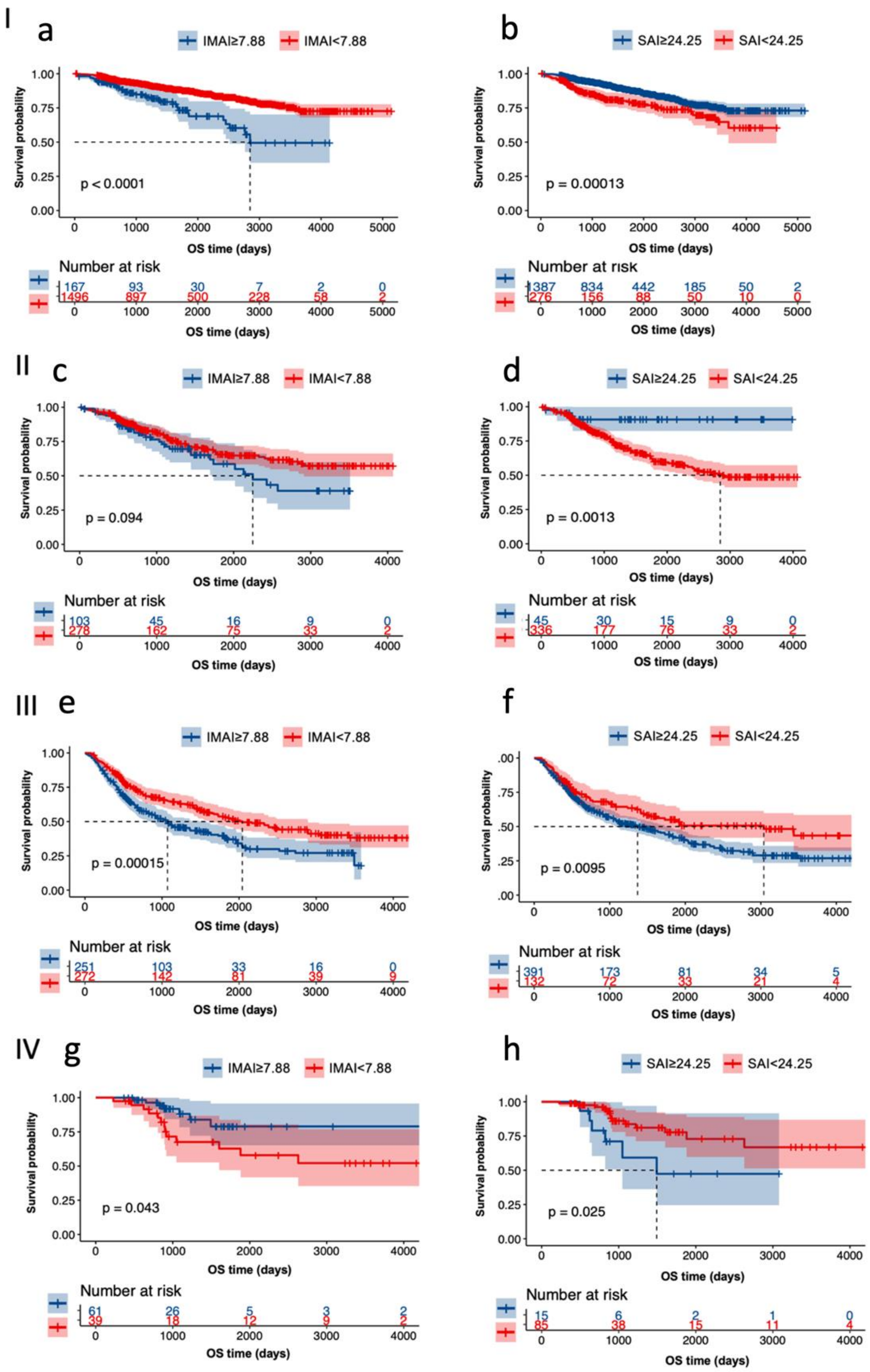

Supplementary Fig. S3 Kaplan–Meier survival curves showing the overall survival according to TNM stage.

a, b IMAI and SAI in the I stage. **c, d** IMAI and SAI in the II stage. **e, f** IMAI and SAI in the III stage. **g, h** IMAI and SAI in the IV stage. OS = overall survival, IMAI = intramuscular adipose index, SAI = subcutaneous adipose index.

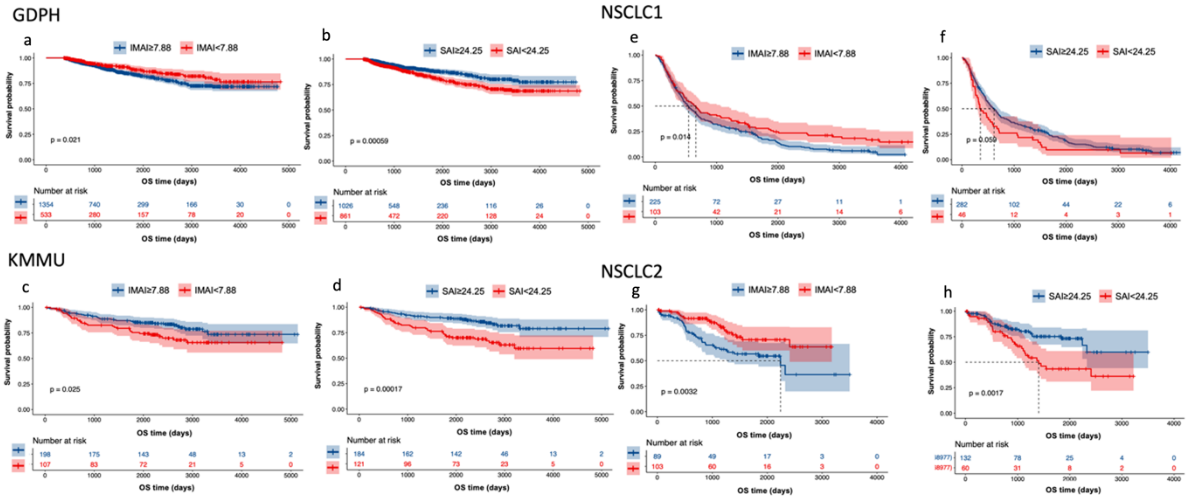

Supplementary Fig. S4 Kaplan–Meier survival curves showing the overall survival according to four centers. a, b IMAI and SAI in the GDPH cohort. **c, d** IMAI and SAI in the KMMU cohort. **e, f** IMAI and SAI in the NSCLC1 cohort. **g, h** IMAI and SAI in the NSCLC2 cohort. OS = overall survival, IMAI = intramuscular adipose index, SAI = subcutaneous adipose index. OS = overall survival, IMAI = intramuscular adipose index, SAI = subcutaneous adipose index.

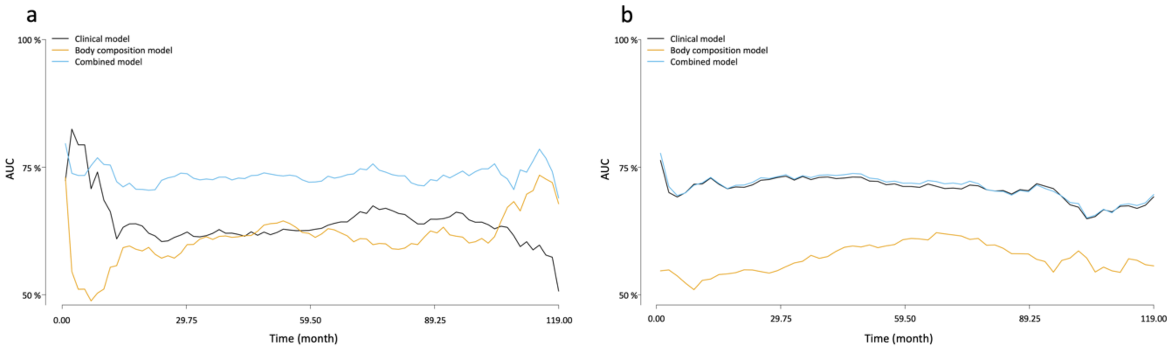

Supplementary Fig. S5 Time-dependent AUC analysis for Cox regression model in overall survival and disease-free survival analysis. a Overall survival. **b** disease-free survival. AUC = area under the curve.
